# Arbuscular mycorrhizal enhancement of phosphorus uptake and yields of maize under high planting density in the black soil region of China

**DOI:** 10.1038/s41598-020-80074-x

**Published:** 2021-01-13

**Authors:** Liyuan Hou, Xiaofei Zhang, Gu Feng, Zheng Li, Yubin Zhang, Ning Cao

**Affiliations:** 1 College of Plant Science, Jilin University, Changchun, 130062 China; 2 Service Center for Agriculture and Rural Development of Hebi, Hebi, 458000 China; 3 College of Resources and Environmental Sciences, China Agricultural University, Beijing, 100094 China

**Keywords:** Fungal host response, Plant symbiosis

## Abstract

Arbuscular mycorrhizal (AM) symbioses are an attractive means of improving the efficiency of soil phosphorus (P) that difficult to be used by plants and may provide a sustainable way of maintaining high yields while reducing P applications. However, quantifying the contribution of indigenous AM fungi on phosphorus uptake and yields of maize (*Zea mays* L.) under field conditions is not particularly clear. Mesh-barrier compartments were applied to monitor the distribution of hyphal P uptake throughout the experimental period under different planting densities and soil depths, over two consecutive years. AM symbioses enhanced plant P-acquisition efficiency, especially during the silking stage, and hyphae of AM fungi was assessed to contribution 19.4% at most to total available P content of soil. Moreover, the pattern of AM depletion of soil P generally matched shoot nutrient demand under the high planting density, which resulted in significantly increased yield in 2014. Although the hyphal length density was significantly decreased with soil depth, AM fungi still had high potential for P supply in deeper soil. It demonstrates the great potential of indigenous AM fungi to maize productivity in the high-yield area of China, and it would further provide the possibility of elimination P fertilizer applications to maintain high yields.

## Introduction

High-density planting systems (more than 70,000 plants ha^−1^) represent one of the most efficient ways of achieving high maize productivity, and is widely applied globally, however, at the expense of high input of resources, such as chemical fertilizer. Thus, a sustainable management plan for P applies in these farming systems to meet the food requirements of China’s growing population is urgently needed. On the one hand, P is an essential resource that is necessary for plant growth and development. Crop yields are constrained by available P content and, therefore, the application of P fertilizers is necessary to achieve global food security^1,2^. On the other hand, long-term and excessive application of P fertilizer has resulted in substantial P accumulation in the soil, which have led to accelerate the consumption of the finite supply of P from rock and diminish environmental quality through the widespread eutrophication of surface water^3,4^. To enhance the efficient use of P, reduce the environmental damage associated with P fertilizer applications, and yet maintain future food security, one option for sustainable P management is to improve the utilization of the large reserve of residual P in soils left behind by the long-term overapplication of P fertilizer in cultivated areas^5^.

It may be possible to fully exploit the soil P legacy and reduce dependence on P fertilizer inputs by maximizing root/rhizosphere efficiency, such as mycorrhizal symbioses, which promote the efficient uptake of P from soil^6–8^.Arbuscular mycorrhizal (AM) fungi form symbiotic associations with the roots of more than 80 percent of terrestrial plants, including agricultural plants such as maize. Mycorrhizal symbioses can benefit plants and soils in many ways, such as enhancing plant growth and drought tolerance by improving mineral and water uptake, root architecture^9–11^, and check the losses of applied nutrients^12^. Meta-analysis had confirmed that AM fungi have a positive effect on the increment of crop grain yields^13^.

High-density planting systems have been reported to negatively effect the diversity and abundance of AM fungal communities^14,15^, and the morphological characteristics of crop roots, which adversely impact nutrient absorption via the root pathway^16^. Consequently, to understand how AM fungi are associated with P nutrition in well-fertilized, high-density planting systems is a first step toward assessing whether P applications could be reduced to improve the management of intensive cropping systems. However, most of these communities were studied in inoculated ecosystems or in pots under experimental conditions^17–19^. Furthermore, growth depressions are caused by fungal demands for organic carbon from the host plant that outweighs any benefits; it might be produced by P transfer via the fungus. Moreover, Li et al.^20^ found that wheat growth depressions caused by AM associations decreased with increasing plant density in calcareous soil, but not just caused by carbon drain. Here we are wondering that how the mycorrhizal pathway responds to adequate carbon sources, and the contribution of indigenous AM fungi to P acquisition in high-density planting systems under in situ field conditions in the black soil region of China remains unclear.

Thus, the primary objective of this work was to measure AM hyphal P uptake in the field by a modified compartment system, estimate the potential contribution of the indigenous AM fungi to P uptake of maize plants to enhance the final product in the high-yield area of China under high planting density mode.

## Results

### Fungal colonization differ among growth stages under high yield planting density

**Figure 1:**
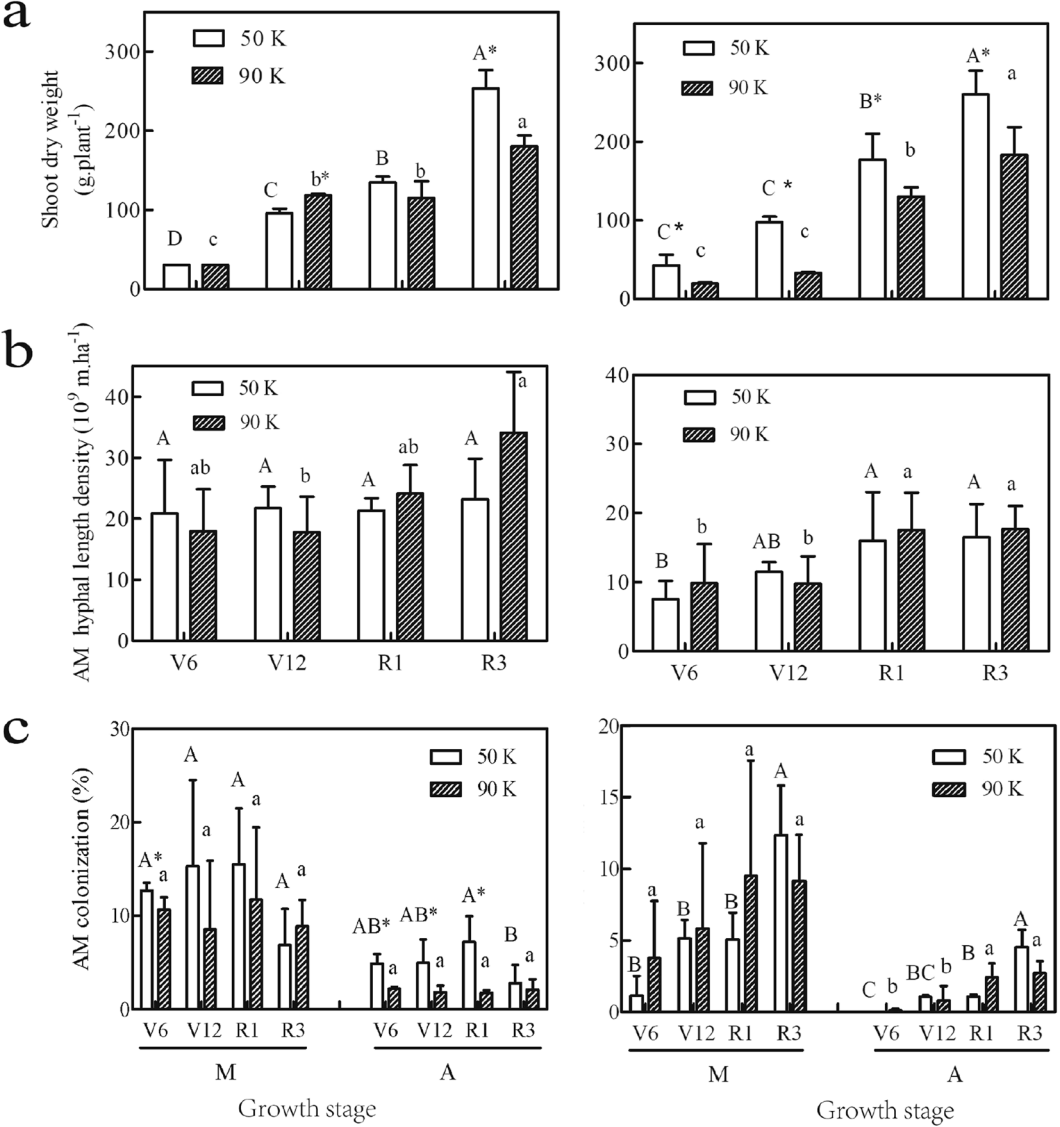
Maize shoot dry weight (**a**), hyphal length density (**b**), and AM fungal colonization (**c**) at different growth stages and planting densities in 2013 (left column) and 2014 (right column). The intensity of mycorrhizal colonization (M) and the arbuscular abundance (A) was measured in the root system. DW, dry weight. Error bars represent SD values of the means (n = 3). Different *uppercase* letters indicate significant differences (*P* $\le$ 0.05) among growth stages under low planting density; different *lowercase* letters indicate significant differences (*P* $\le$ 0.05) among growth stages under high planting density; *asterisks* indicate significant differences (*P* $\le$ 0.05) between different planting densities.

The above-ground biomass (i.e., the shoot biomass), AM fungal density, and mycorrhizal colonization generally increased with the growth stage; however, the amount of increase differed between the cropping years (Fig. 1).

Shoot biomass significantly increased during the vegetative stages, and the average shoot dry weight at the R1 stage was approximately five times higher than that at the V6 stage (Fig. 1a). Hyphal length density gradually increased as maize developed until growth stages R1 to R3 in 2014 (Fig. 1b), whereas no regular distribution during the examined four stages in 2013. Similarly, AM colonization changed apparently with growth stages. The highest infection intensity levels for AM fungi in the root systems of maize plants grown under low and high planting densities were 15.5% and 11.7%, respectively in 2 years (Fig. 1c). Before the R3 stage, the arbuscular abundance of AM fungi in the roots of maize grown under the high planting density significantly increased in comparison to that under the low planting density, especially for the R1 stage, 90 K density was 85.5% higher than 50 K in the 2014 cropping year. Conversely, fungal colonization under 50 K density was pretty high throughout the whole growth period in the 2013 cropping year, and no regular pattern was found against the growth stage.

There was a distinct variation between the 2 years in terms of crop yield. The average grain yield under the high planting density was 16.9% higher than that under the low planting density in 2014. By contrast, a higher yield was obtained under the low cropping density in 2013. However, a comparison of maize yields obtained at the experimental site between 2011 and 2014 under the low- and high-density planting regimes revealed that higher yields were obtained under the high planting density in 2011, 2012 and 2014. The highest yield is 14.1 t ha^−1^, 76.3% higher than the average yield of 8.0 t ha^−1^ obtained in Jilin Province. Moreover, PFPP, which was defined as the yield produced per unit of P applied, was in a high value under both planting densities. The average PFPP under low and high planting density was 238.7 kg kg^−1^ and 269.6 kg kg^−1^, respectively, among four consecutive cropping years (Table 1).

**Table 1:** Effects of planting density on maize yield response and P-use efficiency in the experimental plot during four consecutive cropping years.

Year	2011		2012		2013		2014	
PD	50 K	90 K	50 K	90 K	50 K	90 K	50 K	90 K
Yield	$10.4\pm 0.6$	$12.9*\pm 0.3$	$11.3 \pm 1.2$	$14.1* \pm 1.9$	$14.4 \pm 2.6$	$13.4\pm 2.2$	$10.1 \pm 0.2$	$11.8*\pm 0.0$
PFPP	$214.9 \pm 12.4$	$266.5 \pm 6.2$	$233.5\pm 24.8$	$291.3\pm 39.3$	$297.5\pm 53.7$	$276.9\pm 45.4$	$208.7\pm 4.1$	$243.8 \pm 0.7$

PD, Plant density; 90 K, at a planting density of 90,000 plants ha^−1^; 50 K, at a planting density of 50,000 plants ha^−1^; Yield, crop yield, t ha^−1^; PFPP, the partial factor productivity from applied P, kg yield per kg fertilizer, kg kg^−1^; all data presented are mean values (± SD); asterisk indicates significant difference (*P* $\le$ 0.05) between different planting densities.

### AM fungi enhance shoot P uptake under fast growing stages

**Figure 2:**
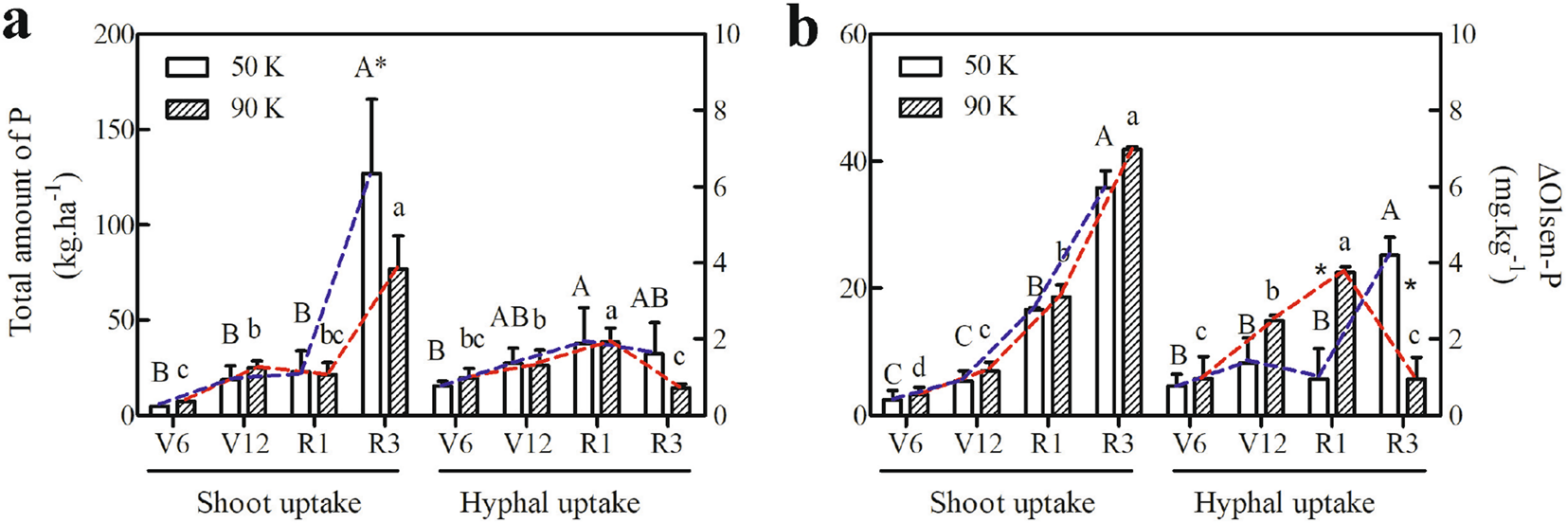
Shoot and hyphal uptake of P ($\Delta$P) at different growth stages and planting densities in 2013 (**a**) and 2014 (**b**). Error bars represent SD values of the means (n = 6). Different *uppercase* letters indicate significant differences in P consumption status (*P* $\le$ 0.05) among growth stages under low planting density; different *lowercase* letters indicate significant differences in P consumption status (*P* $\le$ 0.05) among growth stages under high planting density; *asterisk* indicates significant difference (*P* $\le$ 0.05) between different planting densities.

For the above-ground, plants need plenty of nutrients from the soil to meet their growth. In this study, in general, P uptake increased throughout the growing period regardless of the planting density and cropping year, and levels of increment dependent on its growth period to a large extent. The fastest rate of increase was observed between R1 and R3 stages (*P* $\le$ 0.05), a critical period of impact on final yield and grain quality (Fig. 2). Correspondingly, for the underground, the soil Olsen-P concentration significantly decreased after the R1 stage (*P*$\le$0.05) in the 2013 cropping year. There is no significant difference in soil Olsen-P concentration between the rhizosphere and non-rhizosphere soil (Fig. S1).

Furthermore, two consecutive years of experimental results showed that $\Delta$ Olsen-P ($\Delta$P) value of mycorrhizal hyphae under the high planting density increased significantly during the maize growing period from V6 to R1, which was assumed to be a critical stage for AM colonization of maize under high planting density in this study, however no regularity was found in low planting density. At the R1 stage, in particular, the highest level of hyphal P uptake under the high planting density was recorded in 2014 (3.76 mg kg ^−1^), representing 19.4% of the total available soil P contributed by the mycorrhizal pathway to maize. This indicates that AM fungi played a vital role in the supply of P to the plant during the V6 to R1 stages even though the depletion of soil P by hyphae was inconspicuous. Furthermore, R1 might be a critical stage for the efficient acquisition of P for a dense planting system.

### Variation of the mycorrhizal process in different soil profiles

**Figure 3:**
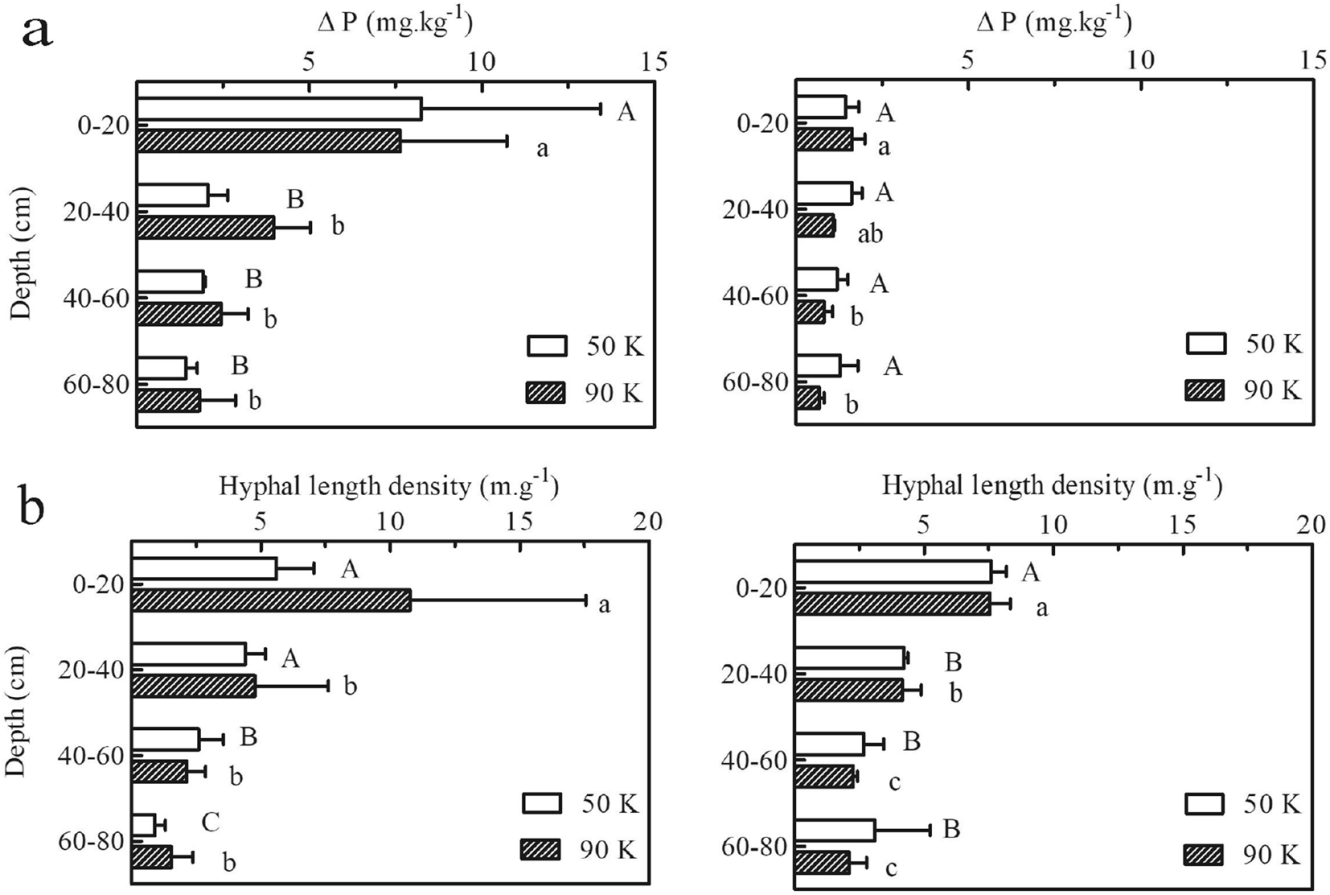
Hyphal P ($\Delta$P) (**a**) and hyphal length density (**b**) at four soil layers ranging from 0–80 cm (20 cm per layer) under different planting densities in 2013 (left column) and 2014 (right column). Error bars represent SD values of the means (n = 3). Different *uppercase* letters indicate significant differences in hyphal P and hyphal length density (*P* $\le$ 0.05) among soil depths under low planting density; different *lowercase* letters indicate significant differences in hyphal P and hyphal length density (*P* $\le$ 0.05) among soil depths under high planting density. There is no significant difference between different planting densities.

Even though $\Delta$P concentration declined with increasing soil depth under both planting densities, the hyphal uptake of P was still extremely deeper in the soil (60–80 cm) (averaged 43% of the value that of at the topsoil layer). Furthermore, the hyphal length density significantly declined with increasing soil depth regardless of the planting density, and the highest hyphal length density values were recorded in the topsoil layer (0–20 cm). Compared with low planting density, hyphal P uptake seemed much more sensitive to soil depth under the high planting density (step down evenly with approximately 10% descent rate) (Fig. 3a). The changing tendency of the hyphal length density with soil depths were accordant basically with that of the hyphal P under both planting densities and cropping years. Plant density did not have a significant effect on the hyphal length density (Fig. 3b).

### Positive relationships between hyphal length density with shoot uptake of P, and hyphal P content

**Figure 4:**
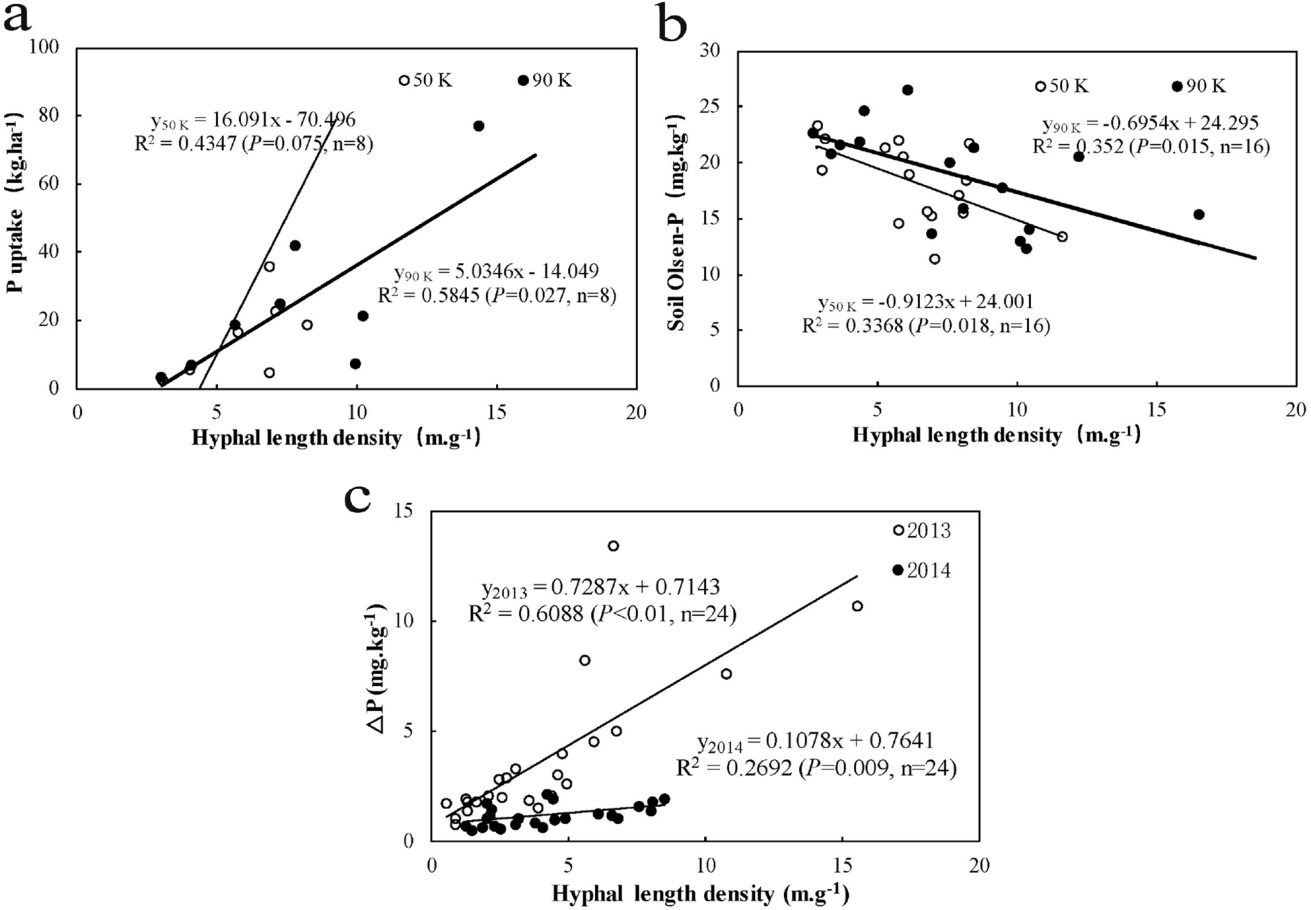
Relationships between AM hyphal length density and P uptake (**a**) and soil Olsen-P (**b**) in the topsoil under different planting densities, and correlation between AM hyphal length density and $\Delta$P in different soil profiles at R3 stage in 2013 and 2014 (**c**) Refer to trail 2).

For the topsoil, although the highest AM fungal colonization rate was only no more than 25% (original data) over the whole experimental period, hyphal length density showed a significant relationship to shoot P content in the high-density planting system, P uptake linearly increased with increasing AM hyphal length density, but no correlation was obtained under the low plant density system (*P* > 0.05) (Fig. 4a). Correspondingly, the soil Olsen-P concentration and the hyphal length density also followed a linear relationship for planting densities. Compared with the values observed at low density, soil Olsen-P content decreased slightly faster with increasing hyphal length density under the high planting density (Fig. 4b). Furthermore, for different soil profiles, the hyphal P uptake and hyphal length density showed an extremely significant positive relationship for both cropping years (*P* < 0.01), and the different slope of the line indicated AM fungi might much more efficient in making soil Olsen-P available in 2013 than in 2014 (Fig. 4c).

## Discussion

Previous work has demonstrated that AM fungi interacting with plants can shift with time within the whole growing season^21–23^. Root infection increased as the growing season progressed and usually peaked in July and August in the maize system^24^. In the present study, hyphal length density per unit area was found to increase in association with the growth of maize slightly, and AM colonization simultaneously increased until the R1 stage, except for individual samples. This is similar to the previous study; the hyphal length density was very low at the V6 stage, increased significantly between V12 and R1, then declined somewhat by R6^25^.

Maize is a mycorrhizal-dependent plant. The extra-rhizosphere mycelium of AM fungi plays an essential role in balancing nutrient demand and supply by highly branched networks in the rhizosphere and absorbing P and organic matter non-rhizosphere soil that cannot be absorbed by plant roots or root hairs^26,27^. AM fungi may also respond strongly to soil fertilization and planting densities^28,29^. Our results confirmed that changes in hyphal P uptake levels during growth coincided with those in the AM colonization rate and the shoot P acquisition by maize, hyphal P which measured by hyphal compartments was increased as the growing period progressed, and peaked at the R1 stage followed by a significant decline at R3 stage under the high planting density. However, it differed under low planting density between two cropping years. This implies that the absorption of P by maize is most dependent on AM fungi in the high-density planting system during the vegetative stages, and R1 is the essential stage at which to measure the contribution of AM fungi to the growth of maize.

Although AM fungi can colonize most terrestrial plants, the colonization rate differs among different crops. Furthermore, it has been dramatically effected by many factors such as soil P level^30^; mycorrhizal colonization was significantly decreased when soil P supply up to over 10 mg kg^−1^ in the wheat plant system^31^. P fertilizers are regularly recommended based on target yields and soil test values to maintain soil fertility and achieve higher crop yields. However, in Northeast of China, low soil temperature during spring inhibits development of maize, due to low root growth and subsequent nutrient acquisition. In practice, some farmers still tend to apply excessive quantities of P to maximize yields and improve soil P fertility. Many studies have shown that the maximum AM colonization rate of maize roots was more than 40% overall growth period^8,24^. In our study, the results from both consecutive years showed that the AM colonization was very low and that the highest infection intensity was no more than 16%. This low infection intensity may due to a high level of basal P supply (16.75 mg kg^−1^ before fertilization) or the usage of chemical fertilizer, pesticide, and tillage practices before this study, such as uniformity of the crop species^32^. Moreover, the colonization of roots by AM fungi is also controlled by shoot P status^10,33^. As we have known, the demand for P nutrient increases gradually with the growth of maize; simultaneously, shoot P concentration decreases with the distribution of the accumulated P to support for the development of various organs, especially at the reproductive stage (see Table S1), which indicates that the amount of seed P reserve is enough for plant growth at the seedling stage, the mycorrhizal pathway is necessary to offset root insufficient absorption since late vegetative stage when the available P of rhizosphere soil is decreased.

**Figure 5:**
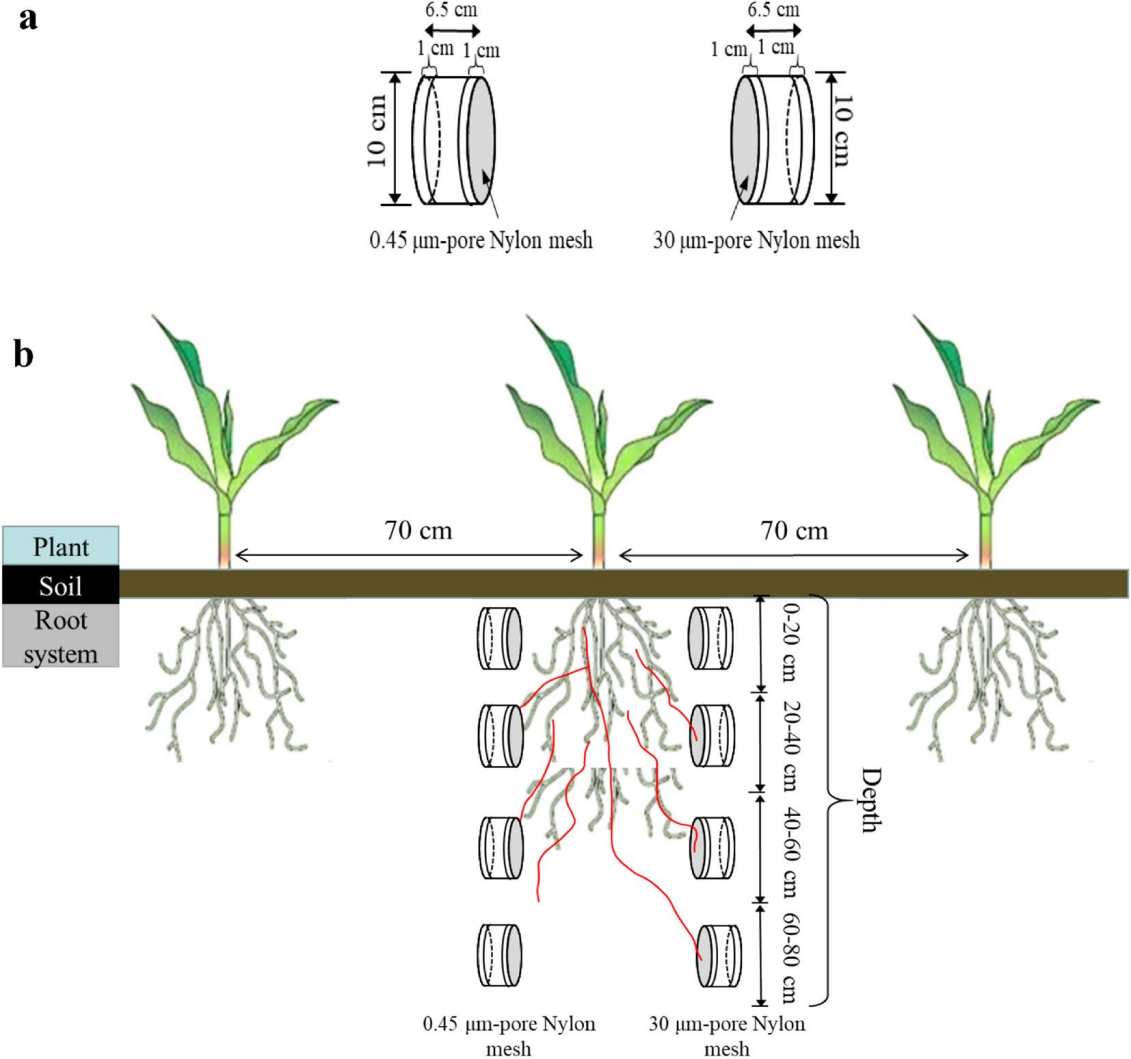
Schematic diagrams of the hyphal compartments (**a**) and the planting system (**b**) used in the experiment. Mycorrhizal hyphae are depicted in red.

Planting density has a severe negative impact on root volume, root surface area, and root length in maize^34^, which can weaken the direct acquisition of nutrients via the roots. Therefore, plant N, P, and K concentrations per plant decreased as the planting density increased, and Shao^35^ reported the total accumulation of P and K per hectare was lower at a density of 90,000 plants ha^−1^ than at a density of 70,000 plants ha^−1^. The availability of soil nutrients, such as the Olsen-P level, had a close association with plant biomass, which could finally affect the crop yield obtained^36^. To maintain yield in an intensively managed planting system, the potential of the mycorrhizal pathway should be deeply involved. The compartment system is well established to quantify the rate of P transport in soil-mycorrhiza systems with the addition of radioactive P isotope as a tracer in various plant specious in pot experiments^37–39^. Very few researches use ^32^P or ^33^P as a tracer and a compartmental design to measure AM phosphorus uptake to estimate the contribution of the indigenous mycorrhizal pathway to total plant P uptake in situ^40,41^. However, because of the radioactivity of phosphorus isotope, safety regulations are highly required in the test process, which significantly limits its application range. To avoid limitations of using radioactive P under field conditions, we used the compartment system with a modified hyphal collection chamber (Fig. 5) to estimate the contribution of AM fungi in high dense planting system by calculating differences of hyphal length density and soil Olsen-P content between two chambers. In our study, the hyphal length density and hyphal P in the different soil profiles were high despite the decrease in abundance of AM fungi with soil depth regardless of planting densities. It might enhance the shoots obtaining and accumulating sufficient P from soil to ensure stable productivity of maize even though the final crop yield obtained under the two planting densities varied between the cropping years 2013 and 2014.

The result of practical production measurement during four consecutive cropping years proved that high planting density could induce high yield; the cropping year 2013 is an exception. Typically, comparing with the impact of planting density, drought, and low soil temperatures at the seedling stage are critical factors affecting maize growth and final yield in Northeast China, meteorological data for the experimental site showed that abundance of precipitation and high soil temperature provided suitable condition for maize seed germination and taking root promptly in the early spring of 2013 (Fig. S2), which shorted seedling period. The significant difference of hyphal P uptake was not observed between the two planting densities during the whole growth period. However, the total amount of P uptake was higher than that of in the cropping year 2014, and the highest increment was up to 254.4% and 83.5% under low planting density, respectively (by comparison of the total amount of P uptake in the R1 stage between 2013 and 2014). Shoot dry weight increased due to plenty of P nutrients. Nevertheless, it significantly increased the lodging rate under high-density growing conditions, especially during the grain-filling stage, which may explain why the highest yields were obtained under the low planting density in 2013.

To further demonstrate that indigenous AM fungi play an essential role in P uptake and transmission in the soil in situ in 2013, control experiments were performed using sterilized soil. As expected, a higher hyphal length density in different soil profiles and higher hyphal P uptake in topsoil were recorded under the high planting density system than under the low planting density. The correlation between the hyphal length density and the hyphal P uptake was extremely significant under dense planting (*P* < 0.01, see Fig. S3). The significant positive relationship between AM hyphal P uptake and hyphal length density suggests that P acquisition in maize from the topsoil mostly relies on root-associated AM fungi, especially for high planting density, because of the formation of a sizeable hyphal network structure in the soil that benefits high-productivity maize crops by facilitating P uptake.

Soil available P is not homogeneously distributed in the vertical soil profile due to the low mobility of P in the soil^42^. Previous studies have reported high levels of AM fungal abundance and species richness in the deep soil layer in arable land by determining the number of species^43,44^ and have shown that the number of AM fungal phylotypes decreased with soil depth, with some phylotypes even observed at depths of up to 100 cm^45^. However, the P uptake efficiency of hyphae in different soil profiles is poorly understood, especially in high-density planting systems. Consistent with most studies, our results showed that although AM fungi were plentiful in the different soil layers, even in deep soil (60–80 cm), the maximum hyphal length density occurred in the topsoil. Even though roots were still readily found in the deep soil layer, the distribution of AM fungi in the soil layers is likely explained by the overall decrease in plant root density with increasing soil depth. The escape of some AM fungi may have caused the abundance of fungi in the deep soil layer into deep soil layers under adverse conditions^44,46^, such as the application of large quantities of fertilizer to the topsoil. Fertilizers can adversely affect AM fungi by inhibiting their growth, development, and function^47^; however, these effects gradually decrease with increasing soil depth. Therefore, there was only a slight (but significant) difference in hyphal P between the surface and deeper soil layers. Furthermore, the hyphal uptake of P and hyphal length density in the different soil profiles showed a strong linear relationship, irrespective of planting density and cropping year, indicating that Olsen-P uptake by maize from the subsoil might also rely on AM fungi because of the limited number of roots and that AM fungi play an immeasurable role in the deep soil.

## Methods

## Experimental site

The experiment was carried out at the Experimental Station of Jilin University, Changchun, China (43$^\circ$56$^\prime$N, 125$^\circ$14$^\prime$E), a typically black soil region, located in the middle of Jilin Province. Fields have been cultivated to grow maize with the plot size of approximately one square kilometer for four consecutive years from 2011 to 2014. The area has a continental monsoon and sub-humid climate, with an annual mean temperature of 5.1$^{\circ }$C and precipitation of 560 mm. The basic soil fertility of experimental site in topsoil was determined as following: pH, 5.99 (water-to-soil ratio of 2.5:1 v/w); organic matter content, 26.3 g kg^−1^; alkali-hydrolyzed nitrogen content, 120.18 mg kg^−1^; total phosphorus content, 0.28 g kg^−1^; and NH_4_Cl-exchangeable potassium content, 140.37 mg kg^−1^. Additionally, soil Olsen-P content in different soil profiles was 16.75  mg kg^−1^, 10.75 mg kg^−1^, 6.95 mg kg^−1^, 6.05 mg kg^−1^, respectively from four soil layers (0 to 80 cm depth, 20 cm per layer).

### Experimental design

The field study was conducted from 2010 to 2014, incorporating four cropping years. In this report, all preliminary experiments were organized in 2013 and 2014 cropping years, referred to as 2013 and 2014 in the context, except for maize yield determination (from 2011 to 2014). Maize (*Zea mays* L. cv. Zhengdan 958) was sown on 1 May 2013 and 30 April 2014 at densities of 50,000 (50 K) and 90,000 plants ha^−1^ (90 K) in both years, with 70 cm row spacing.

The following base fertilizers were applied and mixed into the topsoil by plowing before the maize seeds were sown: 90 kg ha^−1^ as ammonium phosphate, 75 kg ha^−1^ as urea, and 60 kg ha^−1^ as potassium sulfate. As in conventional practice, supplemental urea was top-dressed as 150 kg ha^−1^ between the growth stages of 6-leaf and 12-leaf, to provide an additional N nutrition requirement during plant growth. Pesticides and herbicides were applied as needed according to conventional practice.

To measure AM fungal hyphae in growth, soil hyphal compartments were constructed from PVC chambers and installed in field plots as described by M. Susana Grigera^48^; however, our hyphal compartments were 6.5 cm in height rather than 4.5 cm so that the top and bottom 1 cm of chamber soil could act as a buffer zone to prevent contamination (Fig. 5a). Each pair of hyphal compartments comprised one chamber that was covered with 30-$\upmu$m pore nylon mesh, which allowed penetration by AM fungal hyphae but not plant roots, and another chamber that was covered with 0.45-$\upmu$m pore nylon mesh, which prevented penetration by both hyphae and roots. The inner chambers were filled with 500 g of original soil from the same experimental plot in which the compartment was buried. Three independent trials were designed as following for different aims:

Trial 1: To estimate the contribution of AM fungi during four critical growth stages, hyphal compartments were randomly buried in topsoil, and twelve replicates for each planting density. (Result is shown in Fig. 2).

Trial 2: Maize is a deep-root crop, and roots can extend up to 100 cm in depth. To determine hyphal uptake of P in different soil profiles, the random block design used to determine the effects of the AM fungi in different depths from 0 to 80 cm (20 cm per layer, such as 0–20 cm, 20–40 cm, 40–60 cm, and 60–80 cm) according to the local tillage regimes and plant root distribution range. Then hyphal compartments were buried in the middle of each soil layer in pairs on either side of a maize plant fifteen days after maize emergence (Fig. 5b), three replicates for each treatment. (Result is shown in Fig. 3).

Trial 3: Meanwhile, to eliminate the interaction between AM fungi and bacteria, specific hyphal compartments filled with 500 g of sterilized soil were buried in the field in different depths from 0 to 80 cm in cropping the year 2013. All experimental procedures were the same with trial 2, and soil samples were collected only at milk growth stages (R3) to determine the hyphal length density and hyphal P concentration. (Result is shown in Fig. S3).

### Preparation of samples

Three plants were randomly harvested at the jointing stage (V6), twelfth leaf stage (V12), silking stage (R1), and milk (R3) growth stages in both 2013 and 2014. The plants were cut at the shoot–root junction. Shoots were de-enzymed by heating at 105$^{\circ }$C for 30 min and then dried at 70$^{\circ }$C in an air-drying oven until a constant weight was reached to determine the biomass dry weight (DW, g). Dried shoot samples were roller milled with a pulverizer and sieved through a 0.25-mm mesh before elemental analysis to determine the P content. Roots were carefully dug out, collected rhizosphere soil, washed with tap water to remove residual soil and then stored at 4$^{\circ }$C in a refrigerator while awaiting root colonization analysis. Three pairs of hyphal compartments were randomly removed from the field at the jointing (V6), twelfth leaf (V12), silking (R1), and milk (R3) growth stages, and the top and bottom soil layers (i.e., 1 cm buffer zones) were discarded. Soil samples collected from the surface of the root (rhizosphere soil) and removed from the chamber (non-rhizosphere soil), air dried, sieved through a 1-mm mesh and then divided into two parts, which were used to determine the hyphal density and the Olsen-P concentration. To analyze soil samples from different depths, all of the hyphal compartments were removed from the field between the milk stage (R3) and physiological maturity (R6) growth stages, the rest of the steps were essentially the same above.

To determine maize yield, 9.8 m^2^ of maize (2 rows$\times$7 m length) were harvested from the low- and high-density planting areas at the crop physiological maturity stage (R6), three replicated area was harvested for each planting density. The fresh weight and water content of maize seeds were measured using the conventional method, and the final yield per unit area (kg ha^−1^) was calculated.

### Analysis of P

Shoot P concentration was measured by the molybdo-vanadophosphate method after samples were digested in concentrated H$_2$SO$_4$ and H$_2$O$_2$^49^. Soil available P level was determined as Olsen-P using the molybdo-vanadophosphate method by extracting soil samples with 0.5 M NaHCO$_3$ at pH 8.5^50^. Extraradical hyphae were extracted from each soil sample and stained with Trypan blue. The hyphal length density (HLD) of AM fungi was determined using the grid line intersect method in which AM fungal hyphae were distinguished from non-AM hyphae by the presence of irregular septa, dichotomous branching, irregular wall thickness, and connection to chlamydospores^51^, the net increment of HLD in the rhizosphere was calculated as follow. The reduction was the presence of other soil organisms with which AM fungi interacts:

1 $$\begin{aligned} {Hyphal\ Length\ Density}(HLD) = HLD_{30\upmu \mathrm{m}-pore} - HLD_{0.45\upmu \mathrm{m}-pore} \end{aligned}$$

Where $HLD_{30\upmu \mathrm{m}-pore}$ and $HLD_{0.45\upmu \mathrm{m}-pore}$ represent the soil hyphal length densities of AM fungi in hyphal compartments covered with 30-$\upmu$m and 0.45-$\upmu$m pore mesh, respectively.

The soil samples were collected from the hyphal compartments at each growth stage, and analyzed for $\Delta$Olsen-P (hyphal P or $\Delta$P), which was calculated using the following equation:

2 $$\begin{aligned} C_{\Delta Olsen-P} = C_{Olsen-P0} - C_{Olsen-Px} \end{aligned}$$

Where $C_{Olsen-P0}$ and $C_{Olsen-Px}$ refer to soil Olsen-P values from hyphal compartments covered with 0.45-$\upmu$m and 30-$\upmu$m pore mesh, respectively.

### Measurement of AM fungal colonization

To evaluate AM fungal colonization, the harvested root samples were cut into fragments approximately 1 cm in length. A modified version of the method described by Koske and Gemma^52^ was used to pretreat and stain the root samples. The proportion of the maize root system that was colonized by mycorrhizal fungi was estimated using the method described by Phillips and Hayman^53^. AM fungal colonization is usually presented as infection intensity (M%) and arbuscular abundance (A%), which are calculated by analyzing 30 randomly selected root segments per growth stage using MYCOCAL software (http://www.dijon.inra.fr/mychintec/Mycocal-prg/download.html). And then, we transformed the infection intensity (M%) and arbuscular abundance (A%) to the value of angle using the following equation:

3 $$\begin{aligned} \mathrm{M}= \mathrm{ASIN}(\mathrm{M}\%)*180/ \pi \end{aligned}$$

4 $$\begin{aligned} \mathrm{A}= \mathrm{ASIN}(\mathrm{A}\%)*180/ \pi \end{aligned}$$

Where ASIN represent the Arcsine function to normalize distributions of the infection intensity (M%) and arbuscular abundance (A%).

### Statistical analyses

All data presented are means of untransformed values (±SD), the significance of differences in shoot dry weight, hyphal length density, infection intensity, arbuscular abundance and P content from different growth stages, and hyphal length density and P content from different soil depths between two planting densities was tested using Fisher’s least significant difference (LSD) at *P*$\le$0.05 after a one-way analysis of variance (ANOVA) with IBM SPSS Statistical Version 19.0, IBM Corp, Armonk, NY, USA. Significant differences between low and high planting density for all determined growth stages were compared by t-test at *P*$\le$0.05.Then schematized using GraphPad Prism Version 5.0 (GraphPad Software). Homogeneity of variances is tested using Levene’s Test for Equality of Variances. Results of relationships between hyphal length density with P status were analyzed using Spearman’s rank correlations. The data for AM colonization, including infection intensity and arbuscular abundance of AM fungi, were arcsine transformed to normalize distributions before ANOVA.

## Supplementary information

Supplementary information 1

Supplementary information 2
