# Supplementary material for: Arbuscular mycorrhizal enhancement of phosphorus uptake and yields of maize under high planting density in the black soil region of China
Source: Sci Rep. 2021 Jan 13;11:1100. doi: 10.1038/s41598-020-80074-x (PMC7807008; doi:10.1038/s41598-020-80074-x)
Supplement: Supplementary file 1 — Supplementary information 1 [file 41598_2020_80074_MOESM1_ESM.pdf]

Arbuscular mycorrhizal enhancement of phosphorus uptake and yields of maize under high planting density in the black soil region of China

Liyuan Hou[a], Xiaofei Zhang[b], Gu Feng[c], Zheng Li[a], Yubin Zhang[a]*, Ning Cao[a]*

[a] College of Plant Science, Jilin University, Changchun 130062, China

[b] Service Center for Agriculture and Rural Development of Hebi, Hebi 458030, China

[c] College of Resources and Environmental Sciences, China Agricultural University, Beijing 100083, China

Liyuan Hou and Xiaofei Zhang contributed equally to this work

*Corresponding author: Yubin Zhang; Ning Cao

Addresses: College of Plant Science, Jilin University, 5333 Xi'an Road, Changchun 130062, China.

E-mail: ybzhang@jlu.edu.cn; cao_ning@jlu.edu.cn

Table S1 Dynamic change of shoot P concentration with growth of maize in 2013 and 2014 ($g \cdot kg^{-1}$)

Stage<br>Year<br>Density		2013		2014	
		50 K	90 K	50 K	90 K
**V6**		3.06 ± 0.00	4.01* ± 0.00	2.75 ± 0.41	2.63 ± 0.37
**V12**		3.53 ± 0.23	3.54 ± 0.52	2.58 ± 0.54	2.57 ± 0.66
**R1**		3.33 ± 0.16	2.69 ± 0.61	2.52 ± 0.14	2.26 ± 0.62
**R3**	**Stem and leaf**	2.72 ± 0.30	2.08 ± 0.55	1.05 ± 0.32	1.24 ± 0.29
	**Grain**	2.60 ± 0.52	2.32 ± 1.72	1.61 ± 0.23	1.64 ± 0.33
	**Corncob**	2.36 ± 0.14	2.21 ± 0.50	1.11 ± 0.44	1.01 ± 0.42

Note: 90 K, at a planting density of 90,000 plants $\cdot ha^{-1}$; 50 K, at a planting density of 50,000 plants $\cdot ha^{-1}$; PD, planting density; all data presented are mean values (± SD); *asterisk* indicates significant difference ($P \leq 0.05$) between different planting densities.

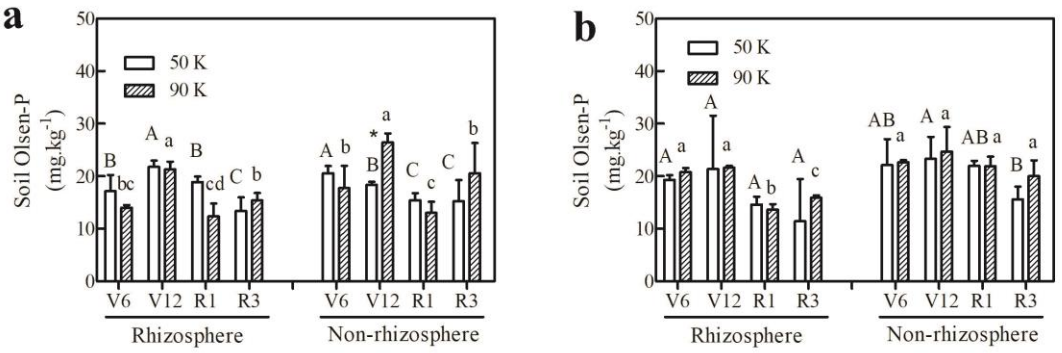

Fig. S1 Changing of Olsen-P concentration in rhizosphere and non-rhizosphere soil during the growth period under two planting densities in 2013 (a) and 2014 (b). Error bars represent SD values of the means (n=3). Different *uppercase* letters indicate significant differences in soil Olsen-P ($P \leq 0.05$) between rhizosphere and non-rhizosphere soil under low planting density; different *lowercase* letters indicate significant differences in soil Olsen-P ($P \leq 0.05$) between rhizosphere and non-rhizosphere under high planting density; *asterisks* indicates significant difference ($P \leq 0.05$) between different planting densities.

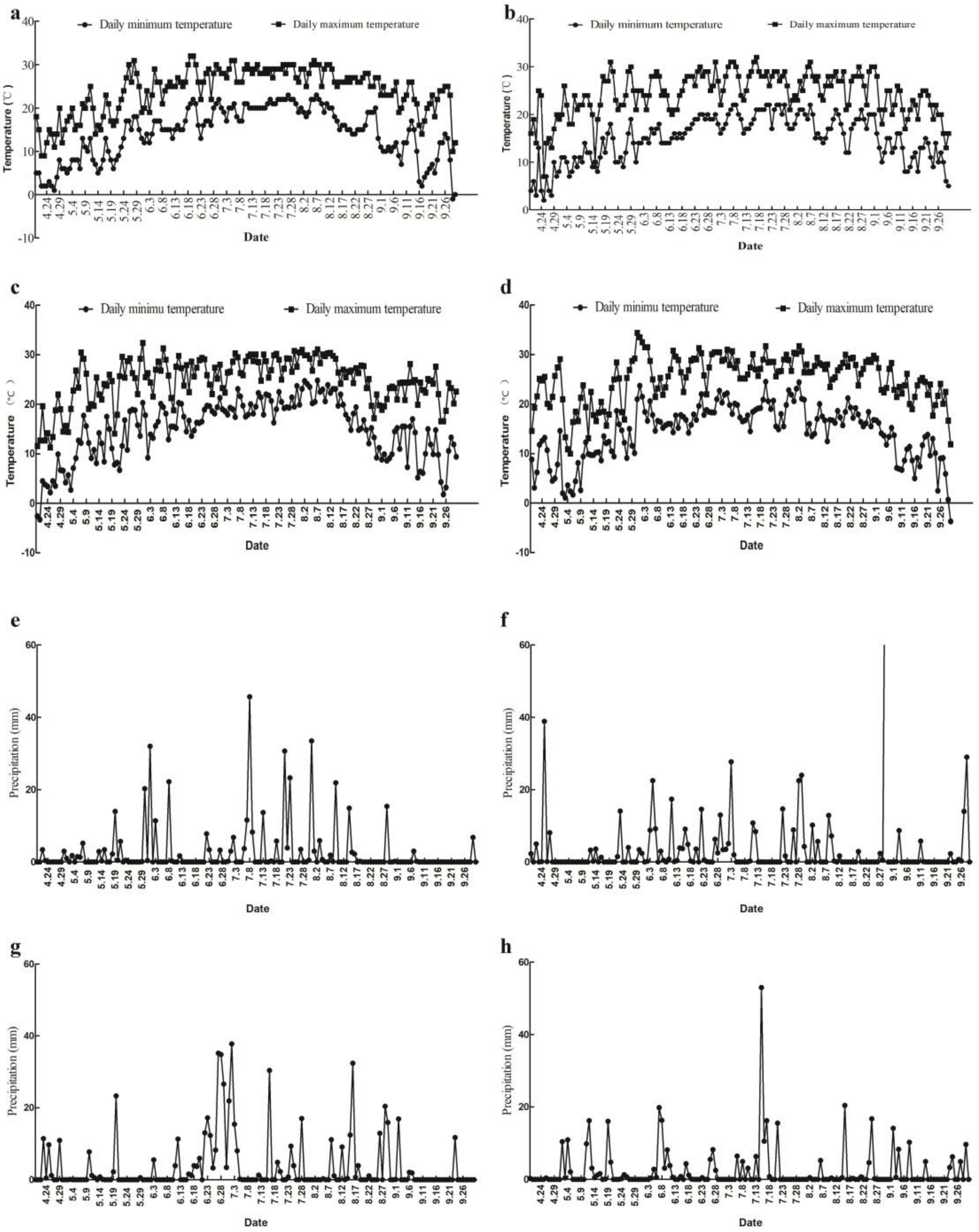

Fig. S2 Daily cumulative temperature and precipitation data of the experimental site during the period of crop growth in 2011 (a, e), 2012 (b, f), 2013 (c, g) and 2014 (d, h)

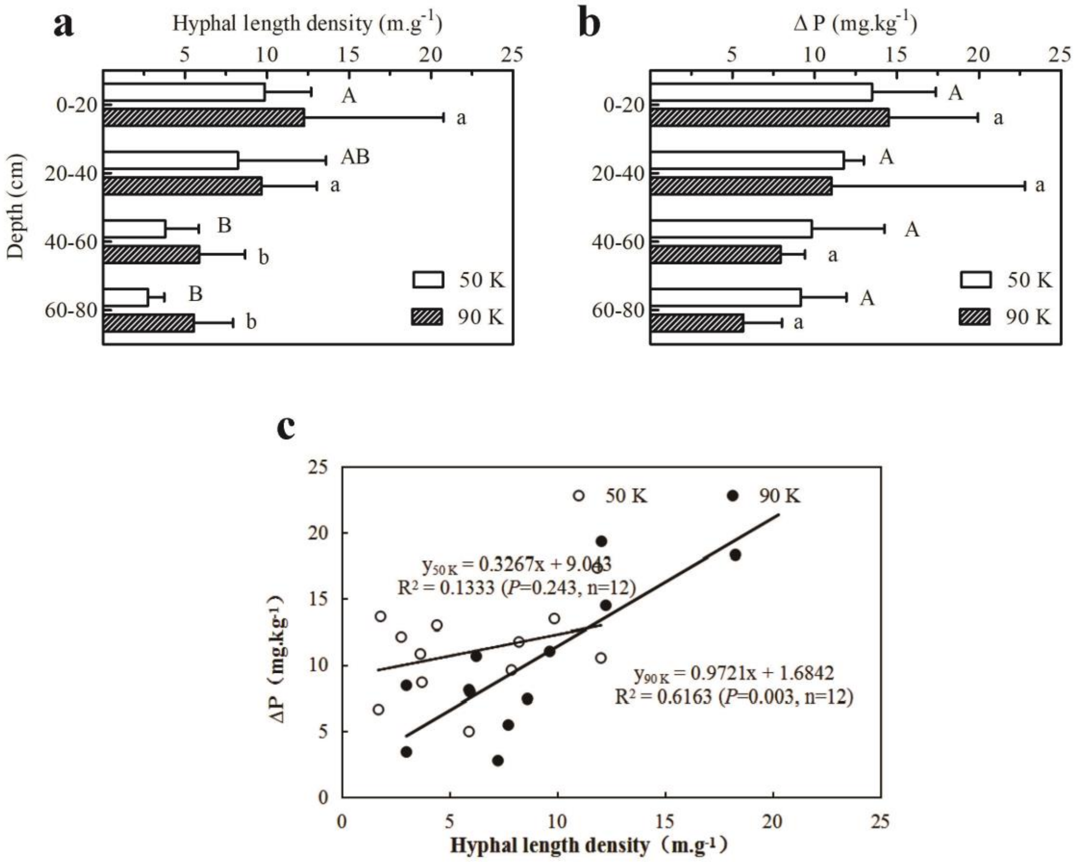

Fig. S3 Hyphal length density (a), hyphal P uptake (ΔP) (b) and relationships between ΔP and AM hyphal length density (c) in different soil profiles at four soil layers ranging from 0-80 cm (20 cm per layer) under different planting densities in 2013. Error bars represent SD values of the means (n=3). Different *uppercase* letters indicate significant differences in hyphal length density and hyphal P ($P \leq 0.05$) among soil depths under low planting density; different *lowercase* letters indicate significant differences in hyphal P and hyphal length density ($P \leq 0.05$) among soil depths under high planting density. There is no significant difference between different planting densities.
