# Supplementary material for: Arbuscular mycorrhizal enhancement of phosphorus uptake and yields of maize under high planting density in the black soil region of China
Source: Sci Rep. 2021 Jan 13;11:1100. doi: 10.1038/s41598-020-80074-x (PMC7807008; doi:10.1038/s41598-020-80074-x)
Supplement: Supplementary file 2 — Supplementary information 2 [file 41598_2020_80074_MOESM2_ESM.doc]

**Arbuscular mycorrhizal enhancement of phosphorus uptake and yields of maize under high planting density in the black soil region of China**

Liyuan Hou^a^, Xiaofei Zhang^b^, Gu Feng^c^, Zheng Li^a^, Yubin Zhang^a^*, Ning Cao^a^*

^a^ College of Plant Science, Jilin University, Changchun 130062, China

^b^ Soil Fertilizer Workstation of Hebi, Hebi 458030, China

^c^ College of Resources and Environmental Sciences, China Agricultural University, Beijing 100083, China

Liyuan Hou and Xiaofei Zhang contributed equally to this work

*Corresponding author: Yubin Zhang; Ning Cao

Addresses: College of Plant Science, Jilin University, 5333 Xi’an Road, Changchun 130062, China. E-mail: ybzhang@jlu.edu.cn; cao_ning@jlu.edu.cn

**Table 1** Effects of planting density on maize yield response and P-use efficiency in the experimental plot during four consecutive cropping years

| **Year** | **2011** | | **2012** | | **2013** | | **2014** | |
| --- | --- | --- | --- | --- | --- | --- | --- | --- |
| **PD** | 50 K | 90 K | 50 K | 90 K | 50 K | 90 K | 50 K | 90 K |
| **Yield /t·ha^-1^** | 10.4±0.6 | 12.9^*^±0.3 | 11.3±1.2 | 14.1^*^±1.9 | 14.4±2.6 | 13.4±2.2 | 10.1±0.2 | 11.8^*^±0.0 |
| **PFPP /kg·kg^-1^** | 214.9±12.4 | 266.5±6.2 | 233.5±24.8 | 291.3±39.3 | 297.5±53.7 | 276.9±45.4 | 208.7±4.1 | 243.8±0.7 |

Note: 90 K, at a planting density of 90,000 plants·ha^-1^; 50 K, at a planting density of 50,000 plants·ha^-1^; PD, planting density; PFPP, the partial factor productivity from applied P; all data presented are mean values (± SD); *asterisk* indicates significant difference (*P*≤0.05) between different planting densities.
